# Acute-onset bilateral myopia induced by hydrochlorothiazide

**DOI:** 10.31744/einstein_journal/2026RC1843

**Published:** 2026-06-23

**Authors:** Luiza Moschetta Zimmermann, José Marcos de Araújo Resende, Heloiza de Castro, Ronaldo Boaventura Barcellos

**Affiliations:** 1 Santa Casa de Misericórdia de São Paulo São Paulo SP Brazil Santa Casa de Misericórdia de São Paulo, São Paulo, SP, Brazil.; 2 Hospital de Olhos Sadalla Amin Ghanem Joinville SC Brazil Hospital de Olhos Sadalla Amin Ghanem, Joinville, SC, Brazil.

**Keywords:** Acute disease, Myopia, Drug-related side effects and adverse reactions, Choroidal effusions, Sulfonamides, Hydrochlorothiazide

## Abstract

Sulfonamides are widely prescribed in clinical practice. Although rare, these have been shown to trigger an idiosyncratic reaction characterized by an acute myopic shift, likely caused by ciliochoroidal effusion and anterior rotation of the ciliary body, resulting in forward displacement of the iris-lens diaphragm. We report the case of a patient who developed blurred vision and acute myopia on cycloplegic refraction after initiating hydrochlorothiazide. The symptoms resolved completely after discontinuation of the medication. Although most of the literature highlights this reaction in association with topiramate, it is important to note that any sulfonamide can potentially lead to a similar clinical presentation.

## INTRODUCTION:

Acute myopia syndrome associated with anterior chamber shallowing is a rare idiosyncratic reaction to certain medications. Although topiramate is the most commonly implicated drug,^(1,2)^ other sulfonamide derivatives have also been associated with similar manifestations.^(3,4)^ The clinical presentation typically includes acute bilateral vision loss, sometimes accompanied by nausea, headache and red eye.^(5)^ On examination, findings may include shallow anterior chambers, conjunctival injection, corneal edema, and retinal folds.^(5)^ Diagnosis is primarily clinical and underscores the importance of reviewing the patient's current medications.^(5)^

The proposed mechanism involves ciliochoroidal effusion and anterior rotation of the ciliary body, leading to a myopic shift.^(6)^ Here, we report a case of acute myopia syndrome induced by hydrochlorothiazide (HCTZ), a commonly prescribed sulfonamide derivative.

## CASE REPORT

A 28-year-old woman presented with acute bilateral visual deterioration lasting one day. She had previously maintained good uncorrected visual acuity (UCVA) and did not require corrective lenses. For the past three weeks, she had been taking HCTZ and betadine for Meniere's disease, an inner ear disorder characterized by recurrent episodes of disabling vertigo, nausea, fluctuating hearing loss, and tinnitus.

**Figure 1:**
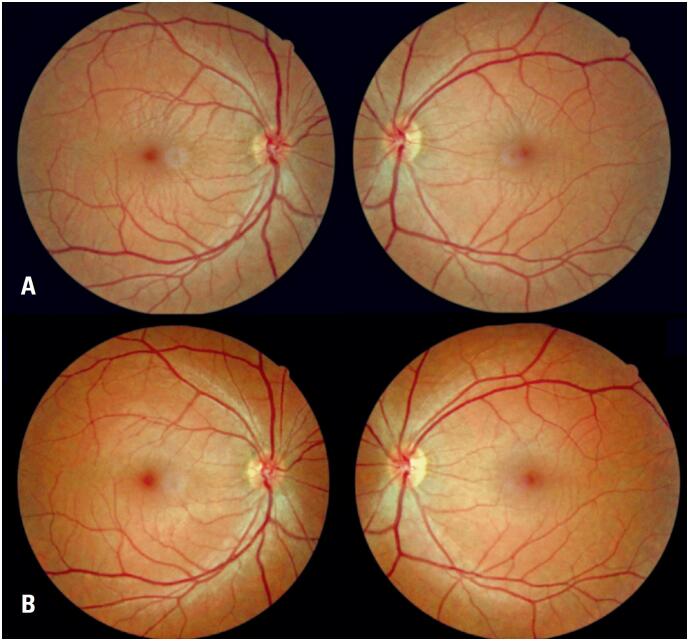
Fundus retinography at initial presentation showing bilateral radiating retinal folds in the macula (A), with improvement after discontinuation of hydrochlorothiazide (B)

On examination, uncorrected visual acuity (UCVA) was worse than 20/400 in both eyes (oculus dexter [OD] and oculus sinister [OS]). Cycloplegic refraction revealed bilateral myopia, with spherical equivalents of -4.0D in each eye (Table 1). After refractive correction, best-corrected visual acuity (BCVA) improved to 20/20 in both eyes. Intraocular pressure (IOP) measured by Goldmann applanation tonometry was 14mmHg in the right eye (OD) and 13 mmHg in the left eye (OS). The anterior segment examination was unremarkable, with no conjunctival chemosis or evidence of anterior chamber shallowing. Foundusexamination revealed bilateral radiating retinal folds in the macula (Figure 1).

**Table 1:** Clinical parameters at presentation and after hydrochlorothiazide cessation

	Eye	UCVA	Refraction
Presentation	OD	<20/400	-4.0D
	OS	<20/400	-4.0D
After HCTZ cessation	OD	20/25	-0.75D
	OS	20/20	-0.75D

A diagnosis of drug-induced myopia was made, and HCTZ was discontinued. Five days later, the patient reported complete resolution of visual symptoms and ocular findings (Figure 1). At follow-up, UCVA was 20/25 (OD) and 20/20 (OS) with best-correct visual acuity (BCVA) 20/20 in both eyes. Refractive testing confirmed a return to the baseline spherical equivalent of -0.75D in both eyes (OU).

The study was approved by the Research Ethics Committee of *Santa Casa de Misericórdia de São Paulo*, CAAE: 94152125.8.0000.5479; # 8.043.580.

## DISCUSSION

Our patient developed a transient myopia shift of 4 diopters following HCTZ intake. As HCTZ was the only medication she was taking and all ocular signs and symptoms resolved after its discontinuation, it was considered the likely causative agent. Although numerous reports describe sulfonamide-induced acute ocular changes, this case is noteworthy for including photographic documentation of radiating retinal folds. The proposed mechanism underlying the acute myopic shift involves ciliochoroidal effusion and anterior rotation of the ciliary body, leading to forward displacement of the iris-lens diaphragm.^(6,7)^ This increases the eye's refractive power, resulting in myopic shift. The associated anterior chamber shallowing and angle narrowing may precipitate angle-closure glaucoma, witch was not observed in our patient. The pathophysiology of ciliochoroidal effusion is believed to result from an idiosyncratic reaction^7^, with some studies suggesting the involvement of prostaglandin-mediated pathways.^(5)^ The primary differential diagnoses includeciliary muscle spasm and primary angle-closure glaucoma. Ciliary spasm, typically associated with iridocyclitis or certain medications (*e.g*., anticholinesterases),^(6)^ was excluded as cycloplegic refraction findings were consistent with manifest refraction. Primary angle-closure glaucoma was considered unlikely given the bilateral presentation, which is uncommon for this condition. Additionally, the presence of characteristic findings such as choroidal effusion and retinal folds further supported a drug-induced etiology. Management requires prompt discontinuation of the causative agent, preferably in coordination with an internist to address potential systemic implications.^(8)^ Notably, laser iridotomy is ineffective, as the underlying mechanism does not involve pupillary block.^(8)^

## CONCLUSION

This case highlights the importance of considering drug-induced ciliochoroidal effusion in the differential diagnosis of acute bilateral myopia. It also emphasizes that even commonly prescribed medications may lead to significant ocular complications. Early recognition and prompt discontinuation of the causative agent are essential to prevent adverse outcomes.

## Data Availability

The data supporting the findings of this study are included within the manuscript.
